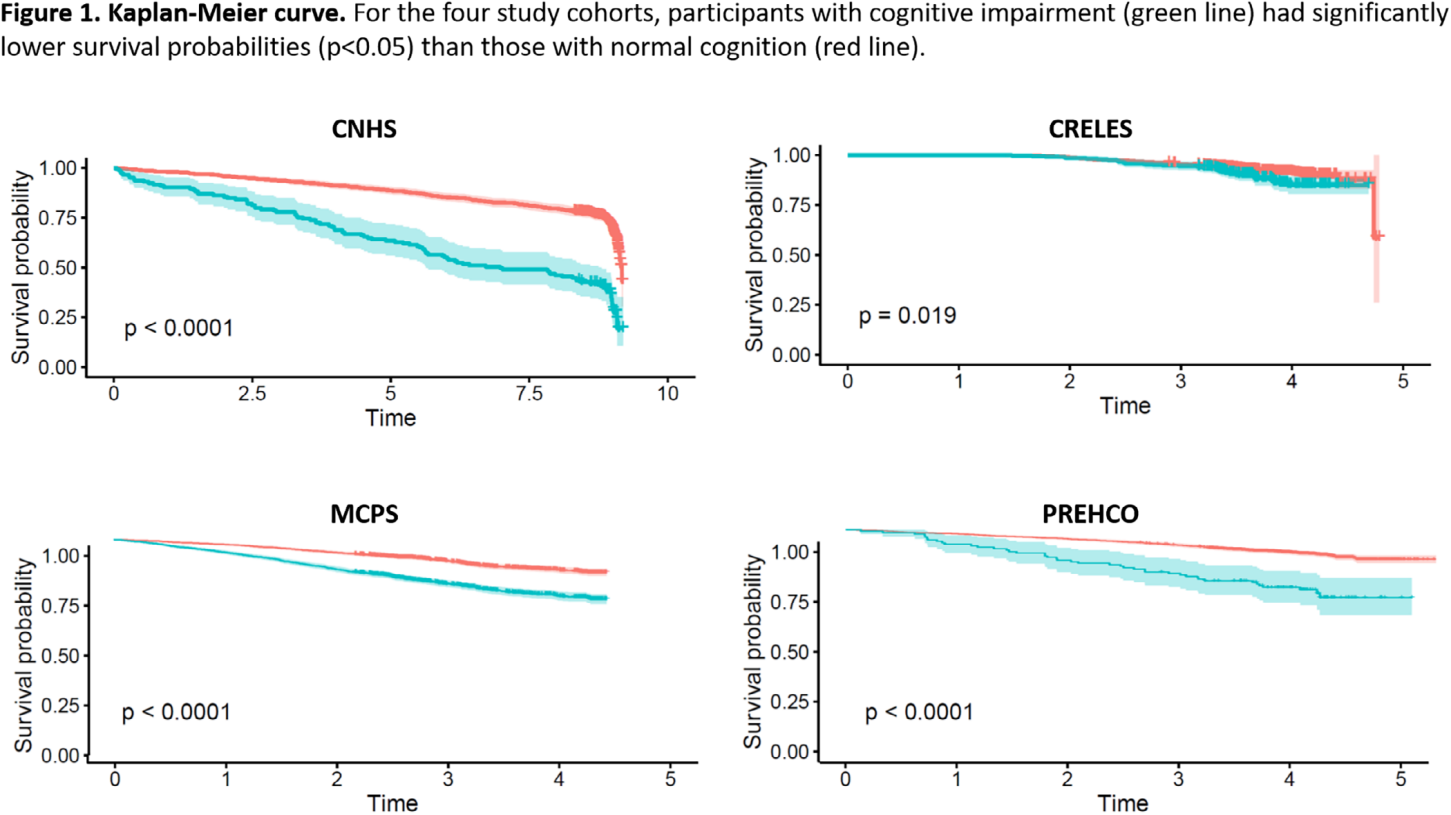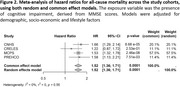# Association Between Cognitive Impairment and Mortality Risk in the elderly: A Longitudinal Study and Meta‐Analysis of four Latin American Cohorts

**DOI:** 10.1002/alz70860_100212

**Published:** 2025-12-23

**Authors:** Paulina Orellana, Carlos Gonzalez‐Carballo, Liset Gonzalez, Carlos Celis‐Morales, Agustin Ibanez, Claudia Duran‐Aniotz, Jesús Alegre, Gabriela Nazar, Diego Aguilar‐Ramirez, Carolina Ochoa‐Rosales

**Affiliations:** ^1^ Latin American Institute for Brain Health (BrainLat), Universidad Adolfo Ibañez, Santiago, Santaigo, Chile; ^2^ Center for Social and Cognitive Neuroscience (CSCN), School of Psychology, Universidad Adolfo Ibañez, Santiago, Chile; ^3^ Experimental Research Unit from the Faculty of Medicine, UNAM, Mexico City, EM, Mexico; ^4^ Latin American Institute for Brain Health (BrainLat), Universidad Adolfo Ibañez, Santiago, Chile; ^5^ Human Performance Lab, Education, Physical Activity and Health Research Unit, University Católica del Maule, Talca, Chile; ^6^ School of Cardiovascular and Metabolic Health, University of Glasgow, Glasgow, United Kingdom; ^7^ Global Brain Health Institute (GBHI), University of California San Francisco (UCSF); & Trinity College Dublin, Dublin, Ireland; ^8^ Latin American Brain Health Institute (BrainLat), Universidad Adolfo Ibañez, Santiago, Chile; ^9^ Psychology Department, Social Sciences Faculty, Universidad de Concepción, Concepción, Chile; ^10^ Latin American Institute for Brain Health (BrainLat), Universidad Adolfo Ibañez, Santiago, Metropolitan Region, Chile

## Abstract

**Background:**

Cognitive impairment (CI) is considered a risk factor for premature mortality. However, it has not been extensively studied in Latin American populations (LATAM). We investigated the association between CI and all‐cause mortality in four LATAM cohorts from Chile, Costa Rica, Mexico and Puerto Rico.

**Method:**

This prospective cohort study included up to *n* = 12,990 participants aged ≥60 years from four studies: the Chilean National Health Survey 2009‐2010 (CNHS *n* = 1,226), the Costa Rican Study on Longevity and Healthy Aging 2004‐2006 (CRELES *n* = 1,898), the Mexico City Prospective Study 2015‐2019 (MCPS *n* = 6,448), and the Puerto Rican Elderly: Health Conditions 2002‐2003 (PREHCO *n* = 3,418). Data on all‐cause mortality was collected until 2020, 2010, 2022, and 2013, respectively. The Mini‐Mental State Examination (MMSE) assessed cognitive performance (higher scores indicate better performance). A CI case was determined according to the MMSE cut‐off criteria established by each cohort. The Kaplan‐Meier estimator and multivariable Cox proportional hazards models were performed. Results were expressed as HR (95%CI) per cohort and in meta‐analysis.

**Result:**

The mean age across the cohorts ranged from 69 years (IQR 64–76) in CNHS to 73 years (IQR 67–81) in CRELES. The follow‐up period varied between 3.69 years in CRELES and 8.88 years in CNHS. The prevalence of cognitive impairment (CI) ranged from 4.5% in PREHCO to 40.9% in MCPS. The mortality rate ranged from 8.9% in CRELES to 34.7% in CNHS. Subjects with CI had a lower probability of survival than those with normal cognition. The risk of mortality associated with cognitive impairment was 66% (HRCNHS=1.66, *p* <0.001), 53% (HRMCPS=1.53, *p* <0.001), and 58% (HRPREHCO=1.58, *p* = 0.01). For CRELES, no significant association was observed (HR=1.22, *p* = 0.25). The meta‐analysis across cohorts yielded a pooled risk of all‐cause mortality of 52% (HR=1.52; *p* <0.001).

**Conclusion:**

LATAM adults aged ≥60 with CI have a lower survival probability and higher mortality risk. Higher MMSE scores are protective against premature mortality. These results are consistent across cohorts and in meta‐analysis.